# Supplementary material for: Distinct effects of complement and of NLRP3- and non-NLRP3 inflammasomes for choroidal neovascularization
Source: eLife. 2020 Dec 11;9:e60194. doi: 10.7554/eLife.60194 (PMC7732340; doi:10.7554/eLife.60194)
Supplement: Transparent reporting form [file elife-60194-transrepform.docx]

***eLife’s* transparent reporting form**

We encourage authors to provide detailed information *within their submission* to facilitate the interpretation and replication of experiments. Authors can upload supporting documentation to indicate the use of appropriate reporting guidelines for health-related research (see [EQUATOR Network](http://www.equator-network.org/%20)), life science research (see the [BioSharing Information Resource](https://biosharing.org/)), or the [ARRIVE guidelines](http://www.plosbiology.org/article/info:doi/10.1371/journal.pbio.1000412) for reporting work involving animal research. Where applicable, authors should refer to any relevant reporting standards documents in this form.

If you have any questions, please consult our Journal Policies and/or contact us: [editorial@elifesciences.org](mailto:editorial@elifesciences.org).

**Sample-size estimation**

- You should state whether an appropriate sample size was computed when the study was being designed
- You should state the statistical method of sample size computation and any required assumptions
- If no explicit power analysis was used, you should describe how you decided what sample (replicate) size (number) to use

Please outline where this information can be found within the submission (e.g., sections or figure legends), or explain why this information doesn’t apply to your submission:

Our detailed and extensive previously published CNV quantifications comparing CNV lesions in *Vegfa^hyper^* mice to those *Vegfa^hyper^* mice lacking inflammasome components (e.g. *Vegfa^hyper^Nlrp3^-/-^* mice) informed sample size requirements for CNV quantifications in this mouse model of AMD (Marneros, 2016, EMBO Mol Med). This information is provided in the METHODS section.

**Replicates**

- You should report how often each experiment was performed
- You should include a definition of biological versus technical replication
- The data obtained should be provided and sufficient information should be provided to indicate the number of independent biological and/or technical replicates
- If you encountered any outliers, you should describe how these were handled
- Criteria for exclusion/inclusion of data should be clearly stated
- High-throughput sequence data should be uploaded before submission, with a private link for reviewers provided (these are available from both GEO and ArrayExpress)

Please outline where this information can be found within the submission (e.g., sections or figure legends), or explain why this information doesn’t apply to your submission:

Each figure and figure legend describes the number of biological replicates used and the statistical approach (described in the Methods section).

**Statistical reporting**

- Statistical analysis methods should be described and justified
- Raw data should be presented in figures whenever informative to do so (typically when N per group is less than 10)
- For each experiment, you should identify the statistical tests used, exact values of N, definitions of center, methods of multiple test correction, and dispersion and precision measures (e.g., mean, median, SD, SEM, confidence intervals; and, for the major substantive results, a measure of effect size (e.g., Pearson's r, Cohen's d)
- Report exact p-values wherever possible alongside the summary statistics and 95% confidence intervals. These should be reported for all key questions and not only when the p-value is less than 0.05.

Please outline where this information can be found within the submission (e.g., sections or figure legends), or explain why this information doesn’t apply to your submission:

All this information is provided in the result section, the figures and figure legends. Exact p-values are shown.

(For large datasets, or papers with a very large number of statistical tests, you may upload a single table file with tests, Ns, etc., with reference to sections in the manuscript.)

**Group allocation**

- Indicate how samples were allocated into experimental groups (in the case of clinical studies, please specify allocation to treatment method); if randomization was used, please also state if restricted randomization was applied
- Indicate if masking was used during group allocation, data collection and/or data analysis

Please outline where this information can be found within the submission (e.g., sections or figure legends), or explain why this information doesn’t apply to your submission:

We describe randomization and blinding to allow for unbiased CNV quantifications in the METHODS section:

***Quantification of the size and number of CNV lesions***

Allocation, treatment and handling of mice were the same across study groups. For all mice randomization was performed and data were collected and processed randomly. Investigators quantifying CNV lesions were blinded to the genotype of the mice. We used 6-weeks-old mice for all CNV quantifications in order to have a uniform age-controlled population of mice in which early stages of CNV lesions are present. ….

Genotyping information was concealed when lesion sizes and numbers were determined to ensure unbiased analysis. Results were subsequently assigned to genotypes and differences between mouse strains were determined. P-values were calculated with a two-tailed unpaired Students t-test. CNV lesion number and average CNV area per mouse are shown (Prism 7.0a, GraphPad). Sample size requirements were determined based on our previous CNV quantifications in *Vegfa^hyper^* mice and those lacking inflammasome components (Marneros, 2016).

**Additional data files (“source data”)**

- We encourage you to upload relevant additional data files, such as numerical data that are represented as a graph in a figure, or as a summary table
- Where provided, these should be in the most useful format, and they can be uploaded as “Source data” files linked to a main figure or table
- Include model definition files including the full list of parameters used
- Include code used for data analysis (e.g., R, MatLab)
- Avoid stating that data files are “available upon request”

Please indicate the figures or tables for which source data files have been provided:
